# The effect of COVID-19 lockdown on psychiatric admissions: role of gender

**DOI:** 10.1192/bjo.2021.927

**Published:** 2021-06-08

**Authors:** Monica Davies, Luke Hogarth

**Affiliations:** Psychiatry, Maidstone and Tunbridge Wells NHS Trust, UK

**Keywords:** Epidemiology, COVID-19, gender, admissions, legal status

## Abstract

**Background:**

The UK went into nationwide lockdown on 24 March 2020, in response to COVID-19. The direct psychiatric effects of this are relatively unknown.

**Aims:**

We examined whether the first UK lockdown changed the demographics of patients admitted to psychiatric hospitals (to include gender, legality, route of admission and diagnoses), independent of seasonal variation..

**Method:**

We conducted an anonymous review of psychiatric admissions aged ≥18 years in the 6-month period after the announcement of the first UK lockdown (March to August 2020), and in the previous year (March to August 2019), in Kent and Medway NHS and Social Care Partnership Trust in-patient facilities. The number of admissions were compared, along with factors that may help to explain the psychological effects of national lockdown.

**Results:**

There was no significant increase in total number of admissions or the gender percentage. However, there was a 11.8% increase in formal sectioning under the Mental Health Act 1983. This increase was sustained and statistically significant across all 6 months. A sustained decrease in admissions via the crisis team was also observed as being statistically significant. Separate diagnoses saw changes in percentage of admissions between March and May. The most statistically significant was schizophrenia admissions for men in April (18.7%), and women in March (18.4%).

**Conclusions:**

Our findings highlight the effect of COVID-19 on the legal status of psychiatric admissions, and emphasise the importance of having a robust, adaptable and open psychiatric service that caters to the ongoing needs of patients, regardless of government restrictions.

In the UK, the first formal diagnosis of COVD-19 was confirmed on 30 January 2020.^1^ In response, a nationwide lockdown was enforced by the UK Government on 24 March 2020. At this point, confirmed cases had risen to 11 080.^2^ The announcement of lockdown was open-ended; however, relaxed restrictions were introduced on 10 May 2020.^2^

In addition to this unknown duration, people were also faced with the threat of redundancy, working from home, financial struggles, restrictions to socialising and the loss of family members and friends. The news was often populated with references to the detrimental effects of this upheaval on the mental health of the general population. However, it was unclear how and if this was reflected in in-patient psychiatric admissions.

Our aim was to review the adult in-patient psychiatric admissions to Kent and Medway NHS and Social Care Partnership Trust (KMPT) services between March 2020 and August 2020, comparing them with admissions during the same time period in 2019. Gender, legal status, length of admission, previous admissions, route of admission and diagnosis on discharge were analysed to assess if the lockdown period had an effect on psychiatric admissions, and if this effect varied between genders.

## Gender paradox

The gender suicide paradox is well-documented in the literature. It refers to the higher prevalence of female depression and the fact that females are more likely to engage in suicide attempts.^3^ A meta-analysis of 1 million participants, across 30 countries, found that the prevalence of depression in women was 14.4%, compared with 11.5% in men.^4^ However, males experience a higher number of completed suicide deaths,^3^ and by more violent means.^5^ Some of the proposed reasons for the higher rates of successful suicide for males include experiences of stress, likelihood of seeking help and the chosen method of suicide.^3^

Women are reported to have more regular stressful life events, whereas men report more frequent traumatic life events, mainly related to financial stress.^3^ The unprecedented stressors of lockdown could be deemed as ‘traumatic’ rather than ‘regular’, especially as the ‘working day’ changed for the vast majority of UK population; for example, with regards to where work could be conducted, if businesses could remain open, how businesses should support themselves with reduced customer footfall and how to pay staff that were unable to work. With the combination of job insecurity and male vulnerability to financial stressors,^3^ did lockdown negatively predispose our male population to psychiatric illness?

In addition, males have been found to be less forthcoming with regards to engagement with mental health services, or even healthcare professionals in general.^6^ Males are less likely to seek help for depression, substance misuse, physical disabilities and stressful live events, among other things.^6^ A study based on data from a Canadian Community Health Survey found that, among those with mental disorders, females were more likely to talk to others or resort to a change in eating habits in response to stress, whereas males were more likely to avoid other people and drink alcohol.^7^ However, studies into the reasons for this reluctance have yet to adequately explain the processes involved in male help-seeking behaviours.^6^

## Coping mechanisms

Psychiatric admission data may allow us to reflect on how, as a population, we were able to cope with the psychological distress associated with a lockdown. Literature published on coping mechanisms utilised during the pandemic has often focused on individuals in the healthcare profession. One study in New York City found that 48% of healthcare professionals reported depressive symptoms and 57% reported distress during the height of the pandemic, and exercise was the most common coping behaviour used.^8^ Unfortunately, despite physical activity being known to have robust benefits against anxiety and depression,^9^ lockdown limited our access. In the height of the pandemic, indoor gyms were closed, and the UK population was limited to a once-daily outing for physical activity. Those who were most clinically vulnerable and advised to shield had even less opportunities.

Despite this legal barrier to a known coping mechanism, a greater issue could lie with our individual ability to determine when a coping mechanism is needed. It has been proposed that individuals who are unable to differentiate between important and unimportant sources of distress find it harder to determine a solution, regardless of gender.^10^ Lockdown created a focus on the coping mechanisms that could be utilised by the general population, allowing for the wider audience to engage with their personal well-being. However, the often-unspoken aspect of psychiatric well-being was that of our patients with known psychiatric diagnoses.

The pandemic brought its own complications to psychiatric services. One study found that 29% of psychiatric patients had worries about their physical health and 11.8% had moderate-to-severe suicidal ideation.^11^ Multiple public and clinical services were closed, to limit close social contact.^12^ There was significant upheaval to services providing individual support in patients’ own homes and communities, and to the trusted, developed and delicate relationships between care providers and their patients (which were limited to phone consultations). Services must anticipate future outbreaks and develop and implement contingency plans.^11^

## Hypothesis

Our initial hypothesis was of an increase in the proportion of male admissions during this time period. This was formulated based on theories relating to male coping behaviours. Although our hypothesis was to determine if there was a varied response to the pandemic between genders, our data also allowed us to look at admission rates as a whole entity. By comparing reason for admission and route of admission, we aimed to assess the effect of lockdown on a cohort of psychiatric patients.

For a virus we knew relatively little about at the start of lockdown, the lasting effects can be predicted with even less certainty. It is one of the few events of the past century that has affected every person in the UK. To understand its direct effect on physical health is important, especially in treating and preventing its spread. Yet, it is also important to understand its potential detrimental effects on other aspects of health. Equally important, is the ability of our NHS to deal with a pandemic. Viewing the effects retrospectively may allow us to pinpoint service provisions that can be adapted in times of national or global distress, with the hope that support will be provided to those most in need during not only this pandemic, but also inevitable future pandemics.

## Method

Retrospective anonymous data was collected for all in-patient admissions between March to August 2019 and March to August 2020, across the KMPT. This included gender, age, legal status, date of admission, length of stay, previous admission to KMPT in-patient services, number of previous admissions, route of admission and diagnosis on discharge. Legal status, in UK psychiatric terminology, refers to pathway into hospital under the Mental Health Act 1983 (MHA).^13^ Informal patients (i.e. voluntary) agree to be admitted for psychiatric treatment as an in-patient. Formal (i.e. sectioned) patients have been detained under the MHA and are unable to freely leave the hospital.^13^

Route of admission was categorised into three main terms: elective, emergency via the crisis team and other emergencies. Diagnoses were classified by the ICD-10 criteria (codes F0–FX).^14^ Each patient was also given a subcategory classification; however, this was not analysed for this data-set. KMPT record patient diagnosis on discharge. If a patient was still admitted at the time of data collection, the patient was given the diagnosis of ‘null’. Therefore, because data collection took place in late 2020, a higher proportion of 2020 patients had a null diagnosis. Over 90% of admissions in March–May 2020 had been discharged by the time of data collection, therefore diagnoses were only compared in March, April and May in 2019 and 2020.

Data was reviewed on a month-by-month basis to assess if the lockdown had a more immediate or sustained change in type and duration of admission, as well as diagnosis; and to remove confounding factors, such as seasonal variance. Statistical *χ*^2^-test was performed with a 95% confidence interval to calculate differences in rate of admissions between subgroups (Microsoft Excel 2014 for Windows). A *P-*value of <0.05 was considered statistically significant.

Ethical review and approval was not required for the study on human participants, in accordance with the local legislation and institutional requirements. Written informed consent for participation and institutional review board approval were not required for this study, in accordance with the national legislation and the institutional requirements.

## Results

In 2019, there were a total of 1537 new admissions to KMPT between March and August (Table 1). In comparison, 2020 had 1457 admissions, which was a reduction of 5.2%. However, it should be noted that there was a 4.13% reduction in beds available in 2020 (decrease from 85 138 to 81 621).

**Table 1:** Total psychiatric admissions in 2019 and 2020: comparing rates of readmission and legal status

		2019, *n* = 1537 (%)	2020, *n* = 1457 (%)	*P*-value
Total admissions	Total	1537	1457	*P* = 0.13
	Male	830 (54.0)	756 (51.9)	
	Female	706 (45.9)	696 (47.8)	
	X	1 (0.1)	5 (0.3)	
Readmission to KMPT	Total	831 (54.1)	798 (54.8)	*P* = 0.07
	Male	435	406	
	Female	396	387	
	X	0	*5*	
Readmission to KMPT within 90 days	Total	323 (21.0)	328 (22.5)	*P* = 0.32
	Male	153	157	
	Female	170	166	
	X	0	5	
Legal status: sectioning	Total	690 (44.9)	826 (56.7)	*P* < 0.05
	Male	386	430	
	Female	303	394	
	X	1	2	

KMPT, Kent and Medway NHS and Social Care Partnership Trust. ‘X’ being indeterminate/unspecified.

The proportion of male and female admissions was not statistically significant between both years. Male admissions accounted for 54% of 2019 admissions and 52% of 2020 admissions (*P* = 0.13). Similarly, there was no significant change to the percentage of patients who were admitted for the first time or readmitted. In 2019, 54.1% of patients were readmissions, compared with 54.8% in 2020 (*P* = 0.07). There was no significant difference in the percentage that were readmitted within 90 days of their previous discharge (21.0% in 2019 and 22.5% in 2020; *P* = 0.32).

In contrast, there was a statistically significant and sustained increase in the percentage of admissions resulting from formal sectioning (Table 2). This was consistent across all 6 months and both male and female genders. In 2019, 55% of in-patient admissions were informal. However, in 2020, only 43% of admissions were informal, and 56.7% of admissions were a result of formal sectioning under the MHA (*P* < 0.05). When comparing each month directly, April (20% increase; *P* < 0.05) and March (16% increase; *P* < 0.05) saw the largest percentage increases in formal sectioning compared with 2019. The increase in admissions was statistically significant in March to June.

**Table 2:** Percentage of psychiatric admissions as a result of formal sectioning

	Total, *n* (%)	Male	Female	X
March				
2019, *n* = 265	104 (39.2)	64	40	0
2020, *n* = 252	138 (54.8)	67	71	0
*P*-value	*P* < 0.05			
April				
2019, *n* = 232	99 (42.7)	54	44	1
2020, *n* = 193	122 (63.2)	70	52	0
*P*-value	*P* < 0.05			
May				
2019, *n* = 244	117 (47.9)	58	59	0
2020, *n* = 255	147 (57.6)	68	79	0
*P*-value	*P* < 0.05			
June				
2019, *n* = 256	114 (44.5)	69	45	0
2020, *n* = 261	152 (58.2)	79	73	1
*P*-value	*P* < 0.05			
July				
2019, *n* = 276	123 (44.6)	73	50	0
2020, *n* = 260	129 (49.6)	69	59	1
*P*-value	*P* = 0.24			
August				
2019, *n* = 264	133 (50.4)	68	65	0
2020, *n* = 236	138 (58.5)	78	60	0
*P*-value	*P* = 0.07			

"X" being indeterminate/unspecified.

**Fig. 1:**
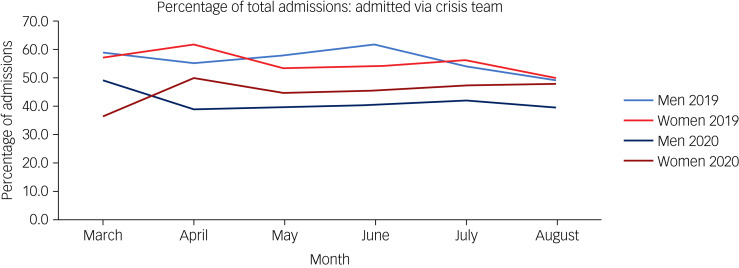
Percentage of total admissions admitted via the crisis team, by gender, in 2019 and 2020.

When comparing admission route, there was a decrease in crisis team admissions for both men and women from 2019 to 2020 (Table 3). Men saw a total decrease from 56.3 to 41.7% (*P* < 0.05), and women saw a total decrease from 55.4 to 45.1% (*P* < 0.05). Figure 1 shows the percentage of admissions per month, categorised by gender.

**Table 3:** Number of admissions via the Crisis team in 2019 and 2020

	Male			Female		
	2019	2020	*P*-value	2019	2020	*P*-value
	*n* = 830	*n* = 756		*n* = 706	*n* = 696	
Total	447 (58.7%)	315 (41.3%)	*P* < 0.05	391 (55.4%)	314 (45.1%)	*P* < 0.05
March	63	62		71	45	
April	67	38		68	47	
May	72	46		64	62	
June	90	59		60	52	
July	86	60		66	55	
August	69	50		62	53	

All confirmed ICD-10 diagnoses were compared with the same month the year prior, with men and women compared separately (Table 4). The largest difference was noted in patients diagnosed with ‘schizophrenia, schizotypal and delusional disorders’ (ICD-10 codes F20–29). A percentage increase of 18.7% (*P* < 0.05) was noted for men from April 2019 to April 2020. Similarly, but more sustained, was the increase in female admissions from 2019 to 2020: there was an increase of 18.4% in March (*P* < 0.05), 14% in April (*P* < 0.05) and 6.9% in May (*P* = 0.19). In contrast, a decrease in percentage of admissions was noted in patients with a formal diagnosis of ‘disorder of adult personality and behaviour’ (ICD-10 codes F60–F69). From 2019 to 2020, female admissions decreased by 5.3% in March (*P* = 0.32) and 12.4% in April (*P* < 0.05); male admissions showed a similar decrease, but with a 1-month delay, decreasing by 7.3% in April (*P* < 0.05) and 5.4% in May (*P* = 0.12). Admissions for ‘neurotic, stress-related and somatoform disorders’ (ICD-10 codes F40–F48) also saw a percentage decrease in 2020: male admissions were down by 7.3% in April (*P* = 0.85) and 5% in May (*P* = 0.08), compared with 2019. Male admissions for ‘mental and behavioural disorders secondary to psychoactive substance use’ (ICD-10 codes F10–F19) also saw a percentage difference in 2020: March and May had a non-significant percentage increase of 5.3% (*P* = 0.30) and 5.5% (*P* = 0.65), respectively, compared with 2019.

**Table 4:** ICD-10 diagnoses given to psychiatric patients admitted in 2019 and 2020

	March, *n* (%)			April, *n* (%)			May, *n* (%)		
	2019	2020	*P*-value	2019	2020	*P*-value	2019	2020	*P*-value
Male									
	*n* = 137	*n* = 123		*n* = 119	*n* = 90		*n* = 122	*n* = 104	
F0	5 (3.6)	8 (6.5)	*P* = 0.29	4 (3.4)	4 (4.4)	*P* = 0.76	4 (3.3)	10 (9.6)	*P* = 0.07
F1	25 (18.2)	29 (23.6)	*P* = 0.30	29 (24.4)	18 (20.0)	*P* = 0.32	32 (26.2)	33 (31.7)	*P* = 0.65
F2	41 (29.9)	37 (30.1)	*P* = 0.99	32 (26.9)	41 (45.6)	*P* ≤ 0.05	34 (27.9)	25 (24.0)	*P* = 0.29
F3	28 (20.4)	23 (18.7)	*P* = 0.72	23 (19.3)	18 (20.0)	*P* = 0.90	21 (17.2)	22 (21.2)	*P* = 0.68
F4	12 (8.8)	10 (8.1)	*P* = 0.85	14 (11.8)	4 (4.4)	*P* ≤ 0.05	11 (9.0)	4 (3.8)	*P* = 0.08
F5	0 (0)	0 (0)		0 (0)	0 (0)		0 (0)	0 (0)	
F6	15 (10.9)	14 (11.4)	*P* = 0.92	14 (11.8)	4 (4.4)	*P* ≤ 0.05	16 (13.1)	8 (7.7)	*P* = 0.12
F7	1 (0.7)	0 (0)	*P* = 0.34	0 (0)	0 (0)		1 (0.8)	0 (0)	*P* = 0.33
F8	5 (3.6)	1 (0.8)	*P* = 0.13	3 (2.5)	1 (11)	*P* = 0.42	2 (1.6)	1 (10)	*P* = 0.60
F9	0 (0)	0 (0)		0 (0)	0 (0)		0 (0)	0 (0)	
FX	5 (3.6)	1 (0.8)	*P* = 0.13	0 (0)	0 (0)		1 (0.8)	1 (10)	*P* = 0.96
Female									
	*n* = 123	*n* = 121		*n* = 110	*n* = 91		*n* = 120	*n* = 130	
F0	4 (3.3)	3 (2.5)	*P* = 0.71	5 (4.5)	5 (5.5)	*P* = 0.80	*2* (1.7)	4 (3.1)	*P* = 0.52
FI	15 (12.2)	12 (9.9)	*P* = 0.56	12 (10.9)	9 (9.9)	0.75	16 (13.3)	18 (13.8)	*P* = 0.93
F2	16 (13.0)	38 (31.4)	*P* ≤ 0.05	16 (14.5)	26 (28.6)	*P* ≤ 0.05	23 (19.2)	34 (26.2)	*P* = 0.31
F3	37 (30.1)	27 (22.3)	*P* = 0.16	28 (25.5)	30 (33.0)	*P* = 0.31	31 (25.8)	25 (19.2)	*P* = 0.13
F4	7 (5.7)	9 (7.4)	*P* = 0.59	11 (25.5)	3 (3.3)	*P* = 0.06	8 (6.7)	10 (7.7)	*P* = 0.87
F5	4 (3.3)	4 (3.3)	*P* = 0.99	1 (0.9)	1 (1.1)	*P* = 0.91	2 (1.7)	1 (0.8)	*P* = 0.48
F6	34 (27.6)	27 (22.3)	*P* = 0.32	33 (30.0)	16 (17.6)	*P* ≤ 0.05	36 (30.0)	34 (26.2)	*P* = 0.32
F7	0 (0)	0 (0)		0 (0)	0 (0)		0 (0)	0 (0)	
F8	1 (0.8)	0 (0)	*P* = 0.32	2 (1.8)	1 (1.1)	*P* = 0.66	0 (0)	0 (0)	
F9	0 (0)	0 (0)		0 (0)	0 (0)		0 (0)	0 (0)	
FX	5 (4.1)	1 (0.8)	*P* = 0.10	2 (18)	0 (0)	*P* = 0.19	2 (1.7)	4 (3.1)	*P* = 0.52

## Discussion

### Reduction in beds

The overarching message from the UK Government at the start of lockdown was to stay home, not only to stay safe, but also to help save the lives of others. The collective fear of the unknown coupled with uncertainties about work stability appeared to cause a rise in community anxiety and depression – according to the headlines.^15^ However, the effect on the rate of psychiatric admissions was unclear.

In KMPT, there was a 5.2% reduction in admissions between March and August 2020, compared with 2019. However, this should be taken in the context of available beds. Over the same time period, KMPT saw a 4.13% reduction in beds. Therefore, the decrease in admissions was non-significant. If we were to view a reduction in available beds as an unrelated variable, it is likely that the admissions data in 2019 and 2020 would be similar with regards to gender, diagnosis and route of admission. However, we hypothesise that a change would occur in relation to the legal status of admissions. Formal sectioning of patients would likely remain constant because of its urgency and the fact that it must result in admission. Therefore, a reduction in beds is most likely to result in a reduction in informal admissions, as these patients can be treated more readily in the community. However, the 11.8% increase in sectioning is unlikely to be solely because of a 4.13% reduction in available beds.

In England, March 2020 saw emergency department attendances for acute coronary syndrome drop by 40% compared with March 2019.^16^ There is no public health measure that could explain an improvement in cardiovascular health of this magnitude, in the space of 12 months. Therefore, this drop in attendance is likely to have resulted in an increase in out-of-hospital deaths and long-term complications of myocardial infarction.^16^ Similarly, Scotland saw an average reduction of 40.7% in emergency department attendances^17^ in the first few months of the first COVID-19 lockdown. This avoidance of healthcare services was seen across the board. The long-term consequences of this reduction are likely to affect all healthcare specialities. There is widespread concern among healthcare professionals that there are many patients in the community who are still not attending services,^18^ risking significant mortality and even death.^18^

The decreases seen in physical health conditions appears to be much more pronounced than those seen by KMPT psychiatry. Why would those with severe physical health problems appear to avoid presenting to hospital any more than those with psychiatric health problems? Lockdown may have provoked a spike in psychiatric conditions, counteracting the general reduction in patients seeking help and explaining why admissions did not drop off as sharply as acute coronary syndrome, for example. Conversely, lockdown should not cause a sudden rise in physical health conditions not related to COVID-19, and it is likely that fear of presenting to hospital (and therefore exposing oneself to COVID-19) resulted in a dramatic drop in acute coronary presentations.

### Increased rate of formal admissions (sectioning)

There was an increased rate of sectioning across 2020, affecting both female and male patients. The largest percentage increase of 20%. was seen in April. Comparatively, in 2018–2019 there was an estimated 2% increase in sectioning from the previous year.^19^

A potential reason for this increase could be linked to the reduction in social and community visits during lockdown. Services in the community needed to be adapted to ensure that social distancing rules were adhered to. Unfortunately, this meant that multiple public and clinical services were closed, including shelters, schools and social services.^12^ Telephone consultations suddenly played an important role in community psychiatry services. Closure of social services may have negatively affected discharge destinations, creating issues with obtaining social housing and locations for follow-up appointments.^12^

Lack of insight and capacity indicates the need for formal sectioning under the MHA. A reduction in community visits had the potential to delay the assessment of psychiatric patients. A delay in assessment could subsequently delay the treatment of acute deterioration of psychiatric conditions. Delayed illness recognition can result in a missed window of opportunity for informal admission, with the patient being more vulnerable to a deterioration of insight. It could also be hypothesised that telephone consultations may have negatively affected assessments; for example, when reviewing a patient's appearance, personal hygiene (including smell) and home environment, all of which factor into a mental state examination.

To counter this argument, however, is the established used of telephone consultations in Australia.^20^ Rural Australian communities have relied on telephone consultations for many years,^20^ facilitating improved rural access to psychiatric healthcare for residents with depression and anxiety.^21^ Therefore, the use of telephone consultations in themselves may not be inferior, but instead the inferiority may stem from the lack of familiarity and efficiency during lockdown.

In Vietnam, aggressive social distancing, imposed for COVID-19, acted as a ‘catalyst’ to transform healthcare service delivery.^22^ Fears of unreliability and limited infrastructure^22^ were overshadowed by the immediate demand for a service that was COVID-19-friendly. The shift, born in a time of desperation, could be the motivation needed to accelerated telemedicine-based practice, promoting a more efficient healthcare system.^22^ With the risk of future lockdowns, telephone services should improve with increased use and funding.

### Increase in psychoactive substance admissions for male patients

In the UK, substance misuse is more common for male patients than female patients.^23^ Stress, particularly in the form of loss, isolation and poor support systems, is a known risk factor for the development of addiction and increased vulnerability to addiction relapse.^24^ It has also been found that those trying to recover from substance misuse benefit from social support^25^ – something that could not be provided to the same extent during lockdown.

This reliance or coping mechanism during times of stress should result in an increase in percentage of male admissions for mental and behavioural disorders secondary to substance misuse. However, our findings were not clinically significant. Regardless, it is important that addiction care is reinforced during lockdown.^26^ The goal is to ensure social support is provided in-line with social distancing as support of this nature has a proven benefit.^25^

### Spike in schizophrenia admissions in both male and female patients

Social interaction is a known benefit in the recovery from schizophrenia,^27^ and is a treatment option that would inevitably be affected by lockdown regulations. As well as limited in-person social interaction, the pandemic restrictions may affect daily routine and group activities, which in turn creates time for vulnerable patients to ruminate on negative cognitions, which has the potential to manifest as paranoid thoughts.^27^

A meta-analysis looking at individuals with pre-existing mental illness during historical pandemics found not only an increase in psychiatric symptoms, but also a reduction in the utilisation of psychiatric services during the pandemics.^27^ The 1918 influenza pandemic had a non-significant increase in compulsory psychiatric admissions.^27^ The spread of the virus, and the extent and duration of the lockdown, makes the COVID-19 pandemic different from previous pandemics.^27^

Extreme uncertainty surrounding the virus has been predicted to exacerbate anxiety, sleep disorders, depression and psychotic symptoms.^28^ There is the potential for blurring of lines between conspiracy theories and paranoid delusions. Conspiracy theories are often shared by others, and in the context of an unprecedented global pandemic, there is a risk of delusion-like beliefs.^28^ Self-quarantine measures can even lead to a preoccupation with the virus. These are all thoughts and beliefs that, before 2020, would have been more aligned with psychotic delusions. However, for psychotic symptoms to result in a psychiatric admission, paranoid delusions need to be personal, idiosyncratic and implausible.^28^ ‘Common’ COVID-19 conspiracies alone do not warrant a schizophrenic diagnosis.

Instead, the spike in admissions could be driven by the circumstances created by lockdown; for example, loneliness is a direct threat to psychological health,^28^ and fear of contracting the virus from others may also intensify pre-existing paranoid tendencies and exacerbate suspicion toward others.^29^ Lockdown may support the persecutory representation of others, that is pre-existing in those with known diagnoses of schizophrenia.^29^

### Decrease in anxiety-related admission for male patients

Across the population, fear and anxiety can proliferate during a pandemic.^30^ This anxiety, along with hospitals being a source of virus contraction, would inevitably create a widespread avoidance of healthcare services. Fear and avoidance are not just limited to psychiatric services.^16^ Looking at our data, a decrease in anxiety-related admissions for men could reflect this fear of the virus and not wanting to burden services.^17^

There may be an alternative viewpoint. Initially it was believed that more people would suffer from anxiety, relating to the fear of losing their jobs and suffering financial struggles. However, there may have been a release of stress and pressure in other aspects. It could be argued that aspects such as commuting were improved by lockdown. The UK Government furlough scheme ensured that the majority of wages were paid, and evictions were stalled. This could be argued as a potential decrease in work-related stress.

Vietnam found that during the first couple of months of a nationwide lockdown, rates of depression, anxiety and stress were lower, with higher level of stress only being a secondary consequence of coming into contact with patients with suspected COVID-19.^31^ The plan by the Vietnamese Government to keep infections low, with an early and intense lockdown, appeared to also be beneficial for the mental health of the general population.

### A decrease in personality-related admissions

Within the spectrum of personality disorders, we can expect individuals to react differently to the pressure of lockdown. For example, patients with borderline or histrionic personality disorders have a strong need for emotional and physical proximity with others.^32^ Therefore, we would expect lockdown to trigger suffering secondary to abandonment, rejection sensitivity and paranoid preoccupations.^32^ It may also intensify interpersonal conflicts, with a misinterpretation of distance as disinterest.^32^ Following this, it would be expected that those with pre-existing borderline/histrionic personality disorders would present more frequently to psychiatric services during lockdown. However, data for March to May suggest that admissions were lower.

In comparison, narcissistic and antisocial personality disorders may struggle with lockdown in other ways, by potentially lacking motivation to follow new guidelines, and being at risk of grandiose self-view with a sense of exemption.^32^ With a disregard for the rules, this subgroup of patients could be at higher risk of exposing themselves and subsequently contracting COVID-19.

Cluster C personality traits, on the other hand, are individuals who have anxious feelings. Potentially fearing contagion during a pandemic, we would expect these patients to have a higher adherence to lockdown regulations, to avoid exposure to the virus.^32^ This may provide some support for our initial admissions data at the start of the pandemic.

### Reduction in admissions via the crisis team

Across the board, admissions via the crisis team accounted for the largest proportion of admissions for men and women in 2019 and 2020. An admission via the crisis team is deemed an emergency admission. However, between March and August 2020, the admissions were reduced consistently compared with the corresponding months in 2019. There could be multiple reasons for this.

A reduction in face-to-face reviews in the community could have resulted in more patients presenting to hospital emergency departments. This would still result in an emergency admission, but by ‘other’ means.

Another difference to crisis team admissions was the change to the consent form for informal admissions. During lockdown, the KMPT consent form for informal admissions was updated to include an agreement by patients to stay in their room until their COVID-19 swab results were negative. In a drive to ensure COVID-19 infections were controlled in in-patient psychiatric hospitals, patients were tested on admission. If negative, they were able to use the communal spaces with the other patients. Unfortunately, at the start of lockdown, this result of a COVID-19 swab could take up to 72 h. This addition to the consent form may have influenced a patient's decision on whether to consent to an informal admission, instead maybe favouring the option to continue with community treatment in their own homes.

Conversely, the findings could be confounded by the introduction of the new admissions pathway by KMPT in early 2020. This pathway allows patients to be admitted from emergency departments without involving the crisis team, and so 2020 would inevitably have a reduction in crisis team–related admissions compared with 2019, despite the pandemic. To understand the effect of this new pathway, we would have to review the proportion of admissions in 2021, to see if this reduction is sustained.

In conclusion, with the percentage of male and female admissions being static, our initial hypothesis of lockdown having a more detrimental effect on men has been disproved. However, admissions data has allowed us to look deeper into different aspects of our psychiatric service, with the hope that services can become more robust to unprecedented events.

Unfortunately, because diagnosis was confirmed on discharge, we were only able to compare the first 3 months of lockdown. To presume that the same trends would continue through lockdown is unfounded. Following on from this data-set, we would be interested to see how the latter months of COVID-19 restrictions and subsequent lockdowns affect the psychiatric admissions. With time and easing of restrictions, there is a hope that patients will no longer fear interacting with health services. This could cause a surge in contact. However, this would be welcomed as patients would receive the care they need.

KMPT only covers the county of Kent. COVID-19 cases throughout the country have differed in rates. It will be interesting to see if areas with higher cases and deaths from COVID-19 have seen differing trends in their psychiatric admissions.

The end to the COVID-19 pandemic is still hard to predict. Even more difficult is the long-term prediction of its sequelae. Looking to the future, it is essential that psychiatric services remain open and are adaptable, and that patients have access to care, even during the most difficult of circumstances.

## Data Availability

The data are not publicly available because of the containment of information (including admission dates) that could compromise the privacy of research participants, as per KMPT policy.
